# Takotsubo Cardiomyopathy in a Nonagenarian With Urosepsis

**DOI:** 10.7759/cureus.8763

**Published:** 2020-06-22

**Authors:** Selin Sendil, Isha Shrimanker, Keerthi Yarlagadda, Binita Bhandari, Vinod K Nookala

**Affiliations:** 1 Internal Medicine, University of Pittsburgh Medical Center (UPMC) Pinnacle, Harrisburg, USA

**Keywords:** takotsubo cardiomyopathy, urosepsis, echocardiogram, cardiac catheterization

## Abstract

Takotsubo cardiomyopathy (TCM) is a rare but reversible myocardial left ventricular (LV) dysfunction, which mimics acute coronary syndrome (ACS) without the presence of significant coronary artery disease (CAD). Emotional stressors may include the death of kin or a life-threatening medical diagnosis whereas physical stressors include infections, endoscopic procedures, exacerbation of asthma, or systemic disorders. A 90-year-old female presented to the ED with nausea, intermittent chest heaviness, and generalized weakness for a duration of three days. Her troponin-I was elevated and an electrocardiogram (EKG) showed T-wave inversions in leads V2-V6 and no ST-segment changes. An echocardiogram (ECHO) revealed an ejection fraction (EF) of 35%-40% with anteroapical hypokinesis. She underwent cardiac catheterization showing nonobstructive CAD. She was diagnosed with pan-sensitive Escherichia coli urosepsis and started on ceftriaxone. She improved clinically and was discharged. A repeat ECHO done a month later showed normal EF. Urosepsis-induced TCM has rarely been reported in the literature. Physicians should have a high index of suspicion of TCM in patients with symptoms mimicking ACS in the presence of a physical stressor like an infection. We report the case of TCM, which resulted from a urinary tract infection (UTI) in an elderly female.

## Introduction

Takotsubo cardiomyopathy (TCM) is an acute and reversible left ventricular (LV) dysfunction characterized by LV apical ballooning and hypo- or a-kinesis mimicking the features of an acute coronary syndrome (ACS) [1]. Patients often present with typical clinical features of ACS including chest pain, shortness of breath accompanied by echocardiographic findings, and biomarker profiles; however, the absence of clinically significant coronary artery disease (CAD) suggests TCM [1]. TCM was initially reported in Japan in 1990 but has gained worldwide attention within the scientific community in the past few decades [2]. It is also known as ‘stress cardiomyopathy’ or ‘broken heart syndrome’ as it is often induced by acute emotional stress [1-2]. TCM is also considered as a syndrome because it develops as a result of various diseases [3]. The condition is usually benign and has a self-limiting course, with recovery within days to weeks following removal of the emotional or physical trigger [2-3]. Urosepsis-induced TCM is rarely reported in the literature. Here, we report a case of TCM which resulted from a urinary tract infection (UTI) in an elderly female.

## Case presentation

**Figure 1:**
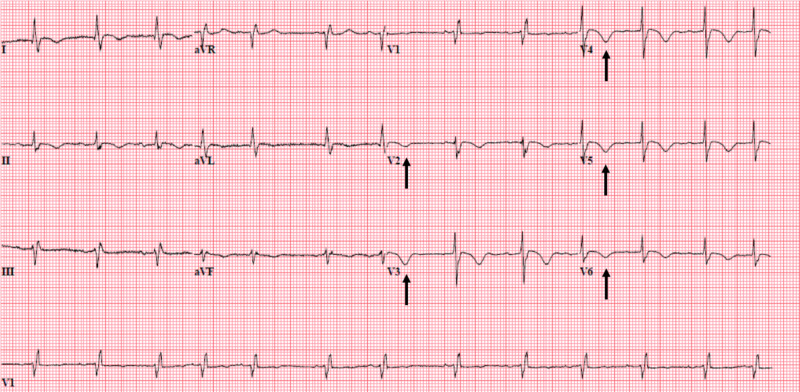
EKG showing T-wave inversions V2-V6 and right bundle branch block with prolonged QT interval. EKG: electrocardiogram

A 90-year-old Caucasian female with a medical history of insulin-dependent type II diabetes mellitus, dementia, essential hypertension, stage III chronic kidney disease, hypercholesterolemia, and former smoking presented to the ED with nausea, decreased oral intake, intermittent chest pain, and generalized weakness for a duration of three days. During the initial presentation, her heart rate was 79/min, blood pressure 178/87 mmHg, oxygen saturation 94% on room air, and temperature 36.7°C. Physical exam revealed bipedal pitting edema and 3/6 systolic murmur in the tricuspid and aortic areas. The remainder of the exam was unremarkable. Her blood work showed a white blood cell count 12 K/uL, neutrophils 81%, lactic acid 1.2 mmol/L, hemoglobin 12.6 g/dL, creatinine 0.97 mg/dL, glucose 208 mg/dL, and troponin-I 0.32 ng/mL. Urinalysis showed 1+ occult blood, 3+ leukocyte esterase, 3+ bacteria, > 50 white blood cells for which she was started on intravenous ceftriaxone. Electrocardiogram (EKG) showed new prominent T-wave inversions from V2-V6 suggesting anterolateral ischemia with no ST-segment changes (Figure 1).

**Figure 2:**
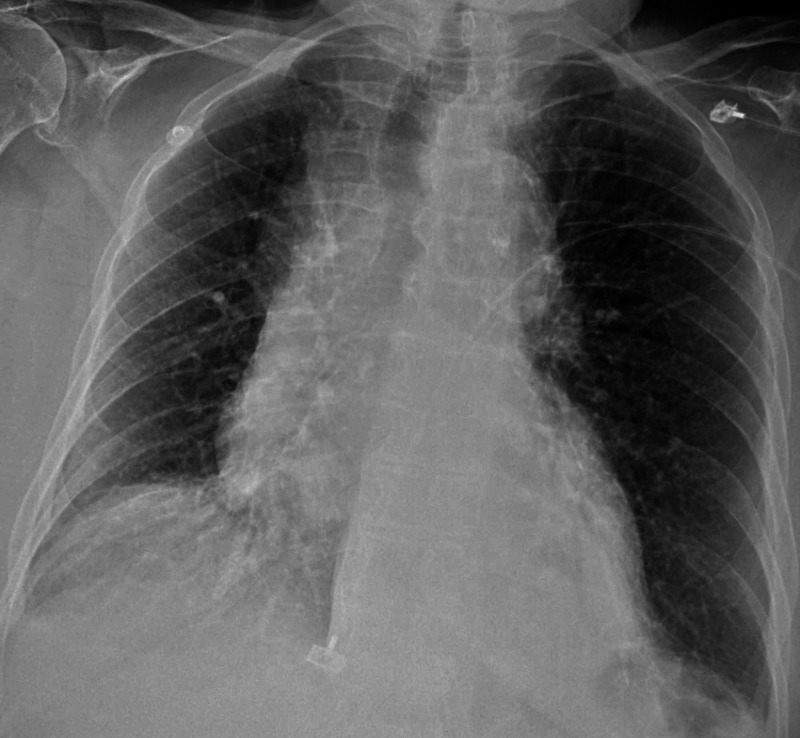
Chest X-ray did not reveal any infiltrate or effusion.

Electrocardiogram also showed right bundle branch block with a prolonged QT interval, which was not a new finding. A chest X-ray did not reveal any infiltrate or effusion (Figure 2).

**Figure 3:**
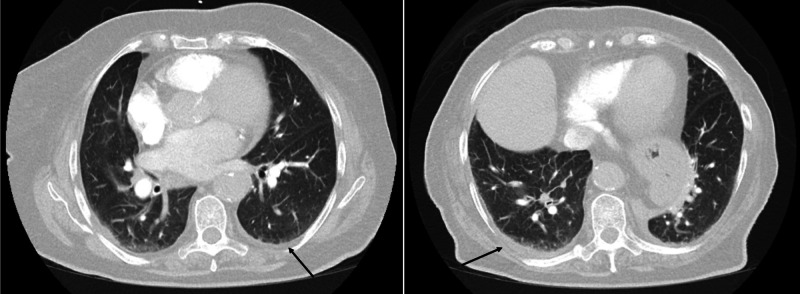
A & B. Different sections of CT of the chest obtained on admission. A & B. Black arrows showing bilateral basal atelectasis and small pleural effusions.

CT scan of the chest did not show pulmonary embolism but revealed small bilateral pleural effusions and mild patchy opacities of the lower lungs, bilaterally, suggesting atelectasis (Figure 3). Urine culture grew pan-sensitive *Escherichia coli* (*E. coli*). She completed the course of antibiotics, showing clinical improvement, and was discharged home five days after admission.

**Figure 4:**
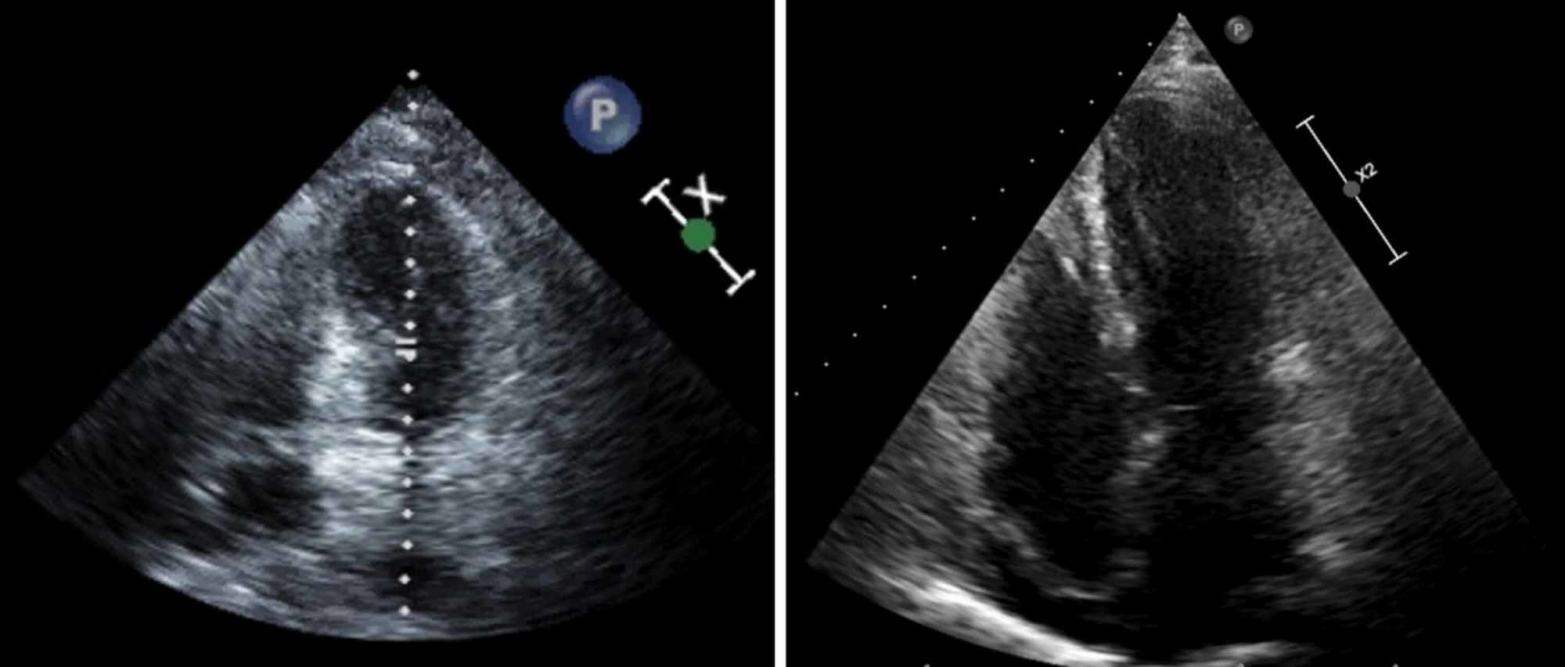
A. ECHO done on admission showing reduced systolic function. B. ECHO done one month later with EF of 60%-65%. ECHO: echocardiogram; EF: ejection fraction

An echocardiogram (ECHO) done on admission showed ejection fraction (EF) of 35%-40% with distal anteroapical wall segment hypokinesis. A repeat ECHO a month later revealed EF of 60%-65% and complete resolution of prior regional wall motion abnormalities of the left ventricle (Figure 4A,B). 

**Figure 5:**
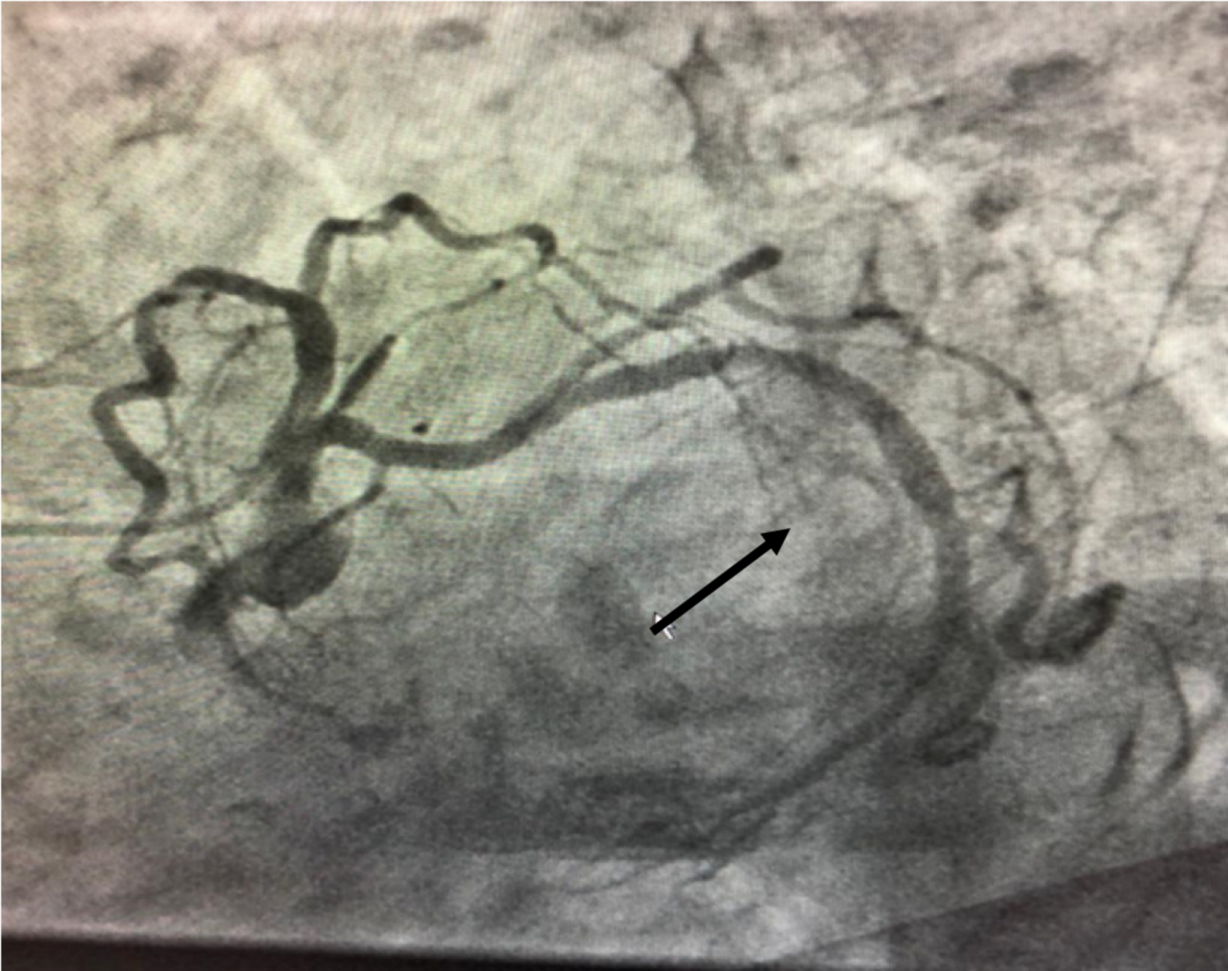
Cardiac catheterization revealing nonobstructive CAD. CAD: coronary artery disease

After extensive discussion regarding goals of care with the patient and the patient’s family, she underwent a cardiac catheterization which revealed nonobstructive CAD (Figure 5).

## Discussion

Takotsubo cardiomyopathy was named after the resemblance of the patient’s LV shape to that of a Japanese octopus trap [1]. Although initially considered rare, with greater awareness and recognition the prevalence is currently reported at 1%-2% of ACS cases presenting to hospitals [1]. The true incidence is likely higher including milder forms that may not receive medical attention and misdiagnosed cases as an ACS. The ECHO findings illustrate regional wall motion abnormalities with a significantly reduced EF. The LV function usually improves over days to weeks [2]. There may be a presence of nonsignificant CAD with TCM; however, this is seen in only 15% of the cases, as 85% have normal coronary arteries [3]. Reversibility of LV changes usually occurs within four to six weeks [3].

Urosepsis-induced TCM has been rarely reported. To our knowledge, there are only a few cases that have been reported in the literature [4-6]. Karvouniaris et al. described a case of TCM following transrectal prostate biopsy leading to pyelonephritis caused by *E. coli* producing extended spectrum β-lactamase [4]. The second case reported by Santoro et al. described acute prostatitis leading to urosepsis caused by *Staphylococcus gallinarum* [5]. The patient was discharged 10 days later, when all EKG anomalies and LV dysfunction recovered (LVEF at discharge >55%). In this report, prostatitis evolved into right epididymitis with scrotal effusion, swelling and pain, and without abscess at CT scan; local inflammation recovered within one month. Both cases had favorable outcomes and successful discharge from the hospital. Omar et al. described a case of TCM triggered by acute pyelonephritis leading to urosepsis [6].

Urinary tract infection is a common diagnosis in the ED and clinical presentation could vary widely especially in the elderly population. As per literature, TCM caused by UTIs happens on rare occasions. The exact pathophysiology of stress cardiomyopathy remains uncertain and several mechanisms may be involved. One of the proposed mechanisms is that a physically or emotionally stressful event (such as a pathogenic microbe, when present with an inflammatory reaction) can lead to increased adrenaline levels by causing a sympathovagal imbalance. A sudden increase in catecholamine levels, which augments the adrenergic activity causes intense coronary vasospasm [7-8]. This vasoconstriction is usually attenuated by central nervous system (CNS) mediated vasodilation [8]. It is unclear as to why mainly the apical region of the heart is affected by sparing basal regions. Though, this can be partially explained by an increased density of adrenergic receptors in the apical region or increased response by apical myocardium to adrenergic stimulation [9]. 

In TCM, LV function returns to normal within a few weeks; however, several complications may occur before the systolic function recovers, and the in-hospital mortality is as high as 5% [10]. Potentially fatal consequences can occur in about 18.9% of cases, which include LV thrombus [10], cardiac tamponade [11], and cardiac rupture [12]. TCM can also lead to atrial and ventricular arrhythmias due to repolarization abnormalities [13-14]. TCM presents with a mortality rate of 3.2% [15]. 

## Conclusions

Timely management after ECHO findings and cardiac catheterization is important. TCM has a clinical resemblance to ACS and thus a coronary angiogram is warranted. TCM is still an uncommon disease and early recognition is vital. Physicians should be aware of the characteristic presentation and consider TCM in the differential.
